# Evaluating the effect of immersive virtual reality technology on gait rehabilitation in stroke patients: a study protocol for a randomized controlled trial

**DOI:** 10.1186/s13063-021-05031-z

**Published:** 2021-01-25

**Authors:** Huihui Cai, Tao Lin, Lina Chen, Huidan Weng, Ruihan Zhu, Ying Chen, Guoen Cai

**Affiliations:** 1 Department of Neurology, Fujian Medical University Union Hospital, Institute of Clinical Neurology, Fujian Medical University, Fuzhou, 350001 Fujian China; 2 Department of Clinical Medicine, Fujian Medical University, Fuzhou, 350001 Fujian China

**Keywords:** Immersive virtual reality, Stroke rehabilitation, Gait rehabilitation, Randomized controlled trials

## Abstract

**Background:**

The high incidence of cerebral apoplexy makes it one of the most important causes of adult disability. Gait disorder is one of the hallmark symptoms in the sequelae of cerebral apoplexy. The recovery of walking ability is critical for improving patients’ quality of life. Innovative virtual reality technology has been widely used in post-stroke rehabilitation, whose effectiveness and safety have been widely verified. To date, however, there are few studies evaluating the effect of immersive virtual reality on stroke-related gait rehabilitation. This study outlines the application of immersive VR-assisted rehabilitation for gait rehabilitation of stroke patients for comparative evaluation with traditional rehabilitation.

**Methods:**

The study describes a prospective, randomized controlled clinical trial. Thirty-six stroke patients will be screened and enrolled as subjects within 1 month of initial stroke and randomized into two groups. The VRT group (*n* = 18) will receive VR-assisted training (30 min) 5 days/week for 3 weeks. The non-VRT group (*n* = 18) will receive functional gait rehabilitation training (30 min) 5 days/week for 3 weeks. The primary outcomes and secondary outcomes will be conducted before intervention, 3 weeks after intervention, and 6 months after intervention. The primary outcomes will include time “up & go” test (TUGT). The secondary outcomes will include MMT muscle strength grading standard (MMT), Fugal-Meyer scale (FMA), motor function assessment scale (MAS), improved Barthel index scale (ADL), step with maximum knee angle, total support time, step frequency, step length, pace, and stride length.

**Discussion:**

Virtual reality is an innovative technology with broad applications, current and prospective. Immersive VR-assisted rehabilitation in patients with vivid treatment scenarios in the form of virtual games will stimulate patients’ interest through active participation. The feedback of VR games can also provide patients with performance awareness and effect feedback, which could be incentivizing. This study may reveal an improved method of stroke rehabilitation which can be helpful for clinical decision-making and future practice.

**Trial registration:**

Chinese Clinical Trial Registry ChiCTR1900025375. Registered on 25 August 2019

**Supplementary Information:**

The online version contains supplementary material available at 10.1186/s13063-021-05031-z.

## Background

Stroke is a serious disease with a high disability rate. Often occurring in elderly populations, stroke-related disability contributes one of the main causes of adult disability [1]. Studies show that stroke survivors experience residual physical dysfunction which has a great impact on their ability to live. Studies have reported that 55–80% of stroke survivors demonstrate continuous motor dysfunction, decreased quality of life, and limited activities in daily life [1–4]. Other studies have reported that 80% of stroke patients experience movement disorders, including loss of balance and gait ability [1, 3]. The disease-related movement disorders and the subsequent decrease in daily living activity can be a great burden to patients, their families, and society. Gait disorder is one of the most common symptoms in stroke sequelae; thus, the recovery of walking ability is the key to improving patients’ self-care ability and quality of life. Compared with that of the healthy people, the gait of patients with cerebral apoplexy often manifests as slowed, shortened standing time on the paralyzed side, too early toes falling when standing, etc. [5]. As such, gait rehabilitation is often the primary goal of stroke rehabilitation [6–8]. As the population continues to age, an increasing number of stroke patients are posed to experience great challenges to disease-related effects. In turn, improving the efficiency of rehabilitation strategies remains of paramount importance.

Virtual reality (VR) is an innovative tool to realize connection, operation, and interaction between human vision and computer-simulated scenarios [9]. Non-immersive VR (for example Xbox Kinect) has been applied in clinical trials of stroke rehabilitation [10, 11]. VR training experience is interesting and enjoyable for the patient, which reduces fatigue, keeps patients in a happy mood, and reduces the boredom of repetitive, conventional rehabilitation. Non-immersive VR-assisted rehabilitation is proposed to provide a more personalized intervention therapy [12]. VR training for stroke patients can therefore improve the participation and autonomy of patients in the rehabilitation process, qualities that have been shown to be more cost and resource effective [13]. Overall, non-immersive VR has been shown to increase limb function learning and improves the quality of life [14].

Recently, immersive VR is a novel VR type. Immersive VR involves a head-mounted display with visual and auditory cues and controllers using haptic (sense of touch) feedback in a 3-dimensional environment [15]. Immersive VR is a technology that provides more realistic environment scene design and object tracking than previous ordinary VR [16], which provides virtual interaction and real-time feedback in vision, touch, hearing, and even motion in realistic scenarios. Patients can experience controllable movement or operation in a simulated virtual environment, so as to achieve the rebuilding or restoring of physical functions.

Immersive VR researches have been reported in the field of pain medicine [17]. In the field of post-stroke rehabilitation, immersive VR has also been reported in upper limb motor function and cognitive ability [16, 18]. However, a few studies have previously explored the application of immersive VR-assisted training in gait rehabilitation after stroke. For example, Biffi et al. found that immersive virtual reality platform enhances the walking ability of children with acquired brain injuries [19] and research by Irene Cortes-Perez has shown that immersive virtual reality improves balance in stroke patients and reduces the risk of falls [20]. Thus, research on immersive VR-assisted training in gait rehabilitation requires its own dedicated investigation. As a VR device of reasonable cost, it has become a powerful research tool for scientific researchers [21, 22]. This study will apply the immersive device to execute VR scenes of rehabilitation training according to clinical practice, so as to systematically evaluate the application of immersive VR technology in the rehabilitation of stroke gait disorders.

## Methods

### Aim

The aim is to assess the effect of immersive VR-assisted rehabilitation training for stroke patients with gait disorders.

### Study design

The study design is a single-blind randomized controlled trial. Other details are described in other sections of the document.

**Fig. 1:**
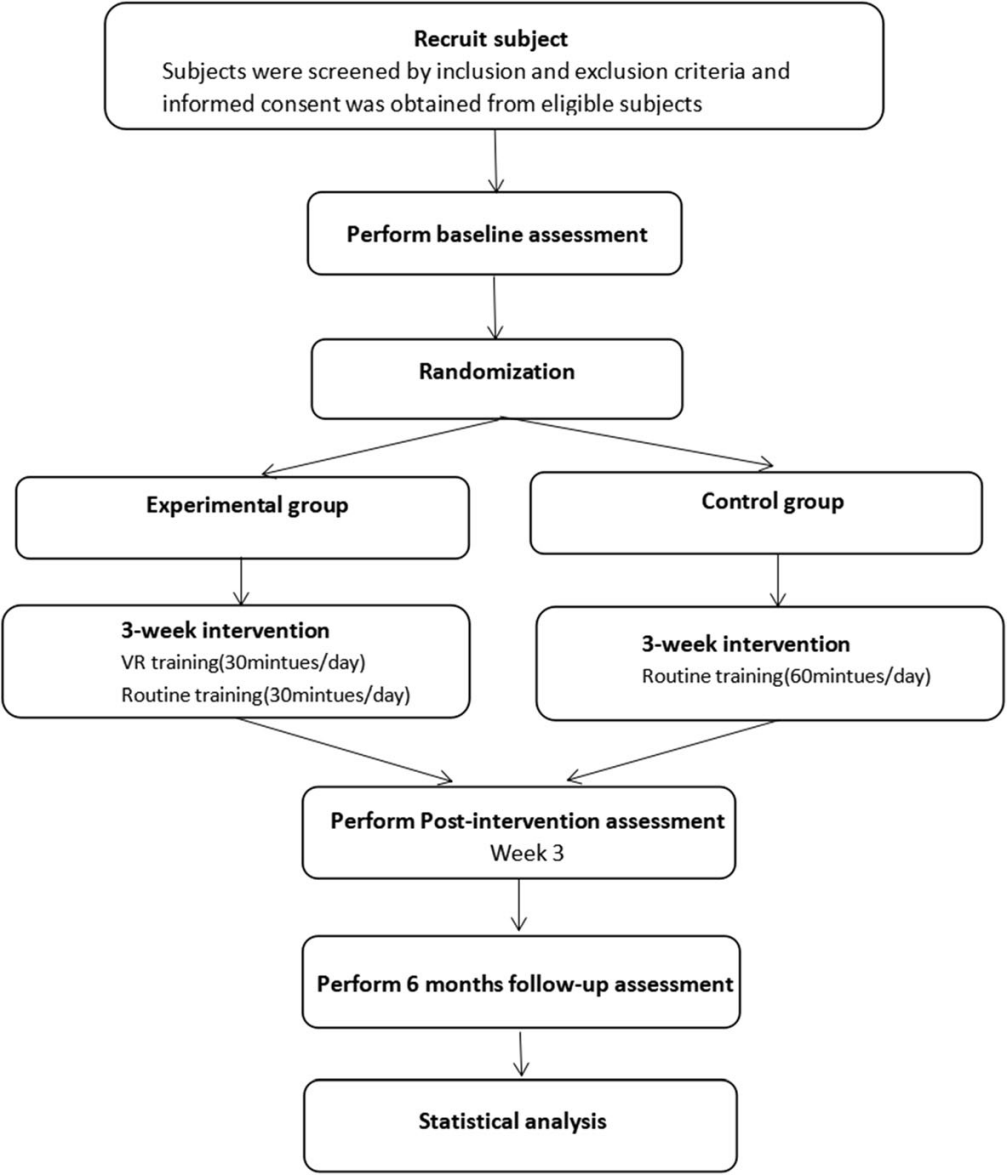
Research flow chart of the experiment

**Fig. 2:**
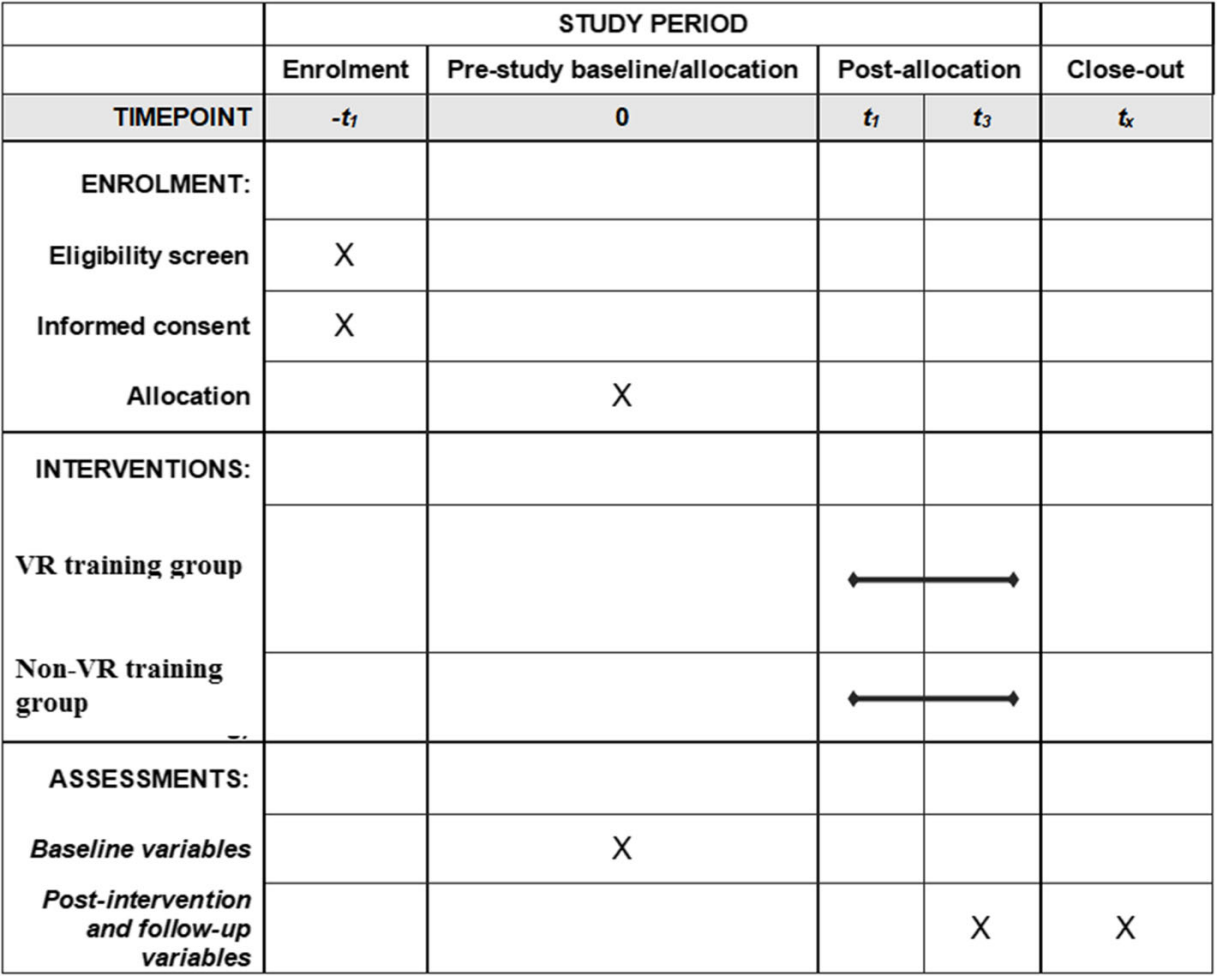
Standard Protocol Items: Recommendations for Interventional Trials (SPIRIT) figure (numbers beside *t* represent weeks)

The protocol will strictly comply with the medical ethics set forth by the medical ethics committee Fujian Medical University Union Hospital. Participants are allocated to the virtual reality gait (VR) training group or the non-virtual reality gait (non-VR) training group. A flow chart of the experimental process is shown in Fig. 1. A checklist of Standard Protocol Items: Recommendations for Interventional Trials (SPIRIT) is provided in Fig. 2.

### Participants

Inclusion criteria: (1) Aged 40–80 years. (2) Patients with ischemic cerebrovascular disease who met the classification and diagnosis criteria of cerebrovascular disease confirmed by head CT or MRI scan [23]. (3) The first ever stroke and initial stroke onset of 1 month or less. (4) Walking dysfunction (the standard of walking dysfunction in stroke patients was patients aged < 60 years with 10-m walking time ≥ 10s; patients aged 60–69 years with 10-m walking time ≥ 12.5 s; patients aged > 70 years with 10-m walking time ≥ 16.6 s). (5) The patient can stand with the help of one person or with crutches and can walk at least l0 m. (6) Lower limb muscle strength level of 3 or above.

Exclusion criteria: (1) Patients with serious behavioral problems or mental disorders. (2) Patients with cognitive impairment (MMSE ≤ 24 points) unable to cooperate with training. (3) Patients with cerebellar stroke, recent lower extremity deep vein thrombosis, quadriplegia, Parkinson’s disease, lower limb fractures, or recent myocardial infarction. (4) Patients with vital organ (heart, lung, liver, kidney, etc.) failure, malignant tumor, or other unstable condition. (5) Patients with history of cerebrovascular accident before this stroke and/or walking dysfunction. (6) Those who have previously completed routine gait training.

### Setting

Rehabilitation training and evaluation of this study will be conducted in the inpatient ward of the Department of Neurology of Fujian Medical University Union Hospital, China.

### Recruitment and consent

The subjects in the trial will be recruited from inpatients in the Fujian Medical University Union Hospital. Their attending physician will brief the patients about the study. If the patients express interest, the physician will provide the patient’s contact information to the research team after obtaining the consent of the patients. The research team will obtain additional information about the patients through interviews and further describe the trial and precautions/benefits to the patients in detail. Additionally, the research team will conduct a qualification examination to verify whether the patients meet the inclusion criteria. If the patient qualifies and agrees to participate in the study, he/she will then be asked to sign the informed consent form and submit the relevant personal written materials. Communication and coordination with patients’ families will occur as necessary. On the consent form, participants will be asked if they agree with the usage of their data should they choose to withdraw from the trial. Participants will also be asked for their permission to share relevant data by the research team with people from the Universities taking part in the research or from regulatory authorities, where relevant. This trial does not involve collecting the biological specimens for storage.

### Randomization and blinding

The eligible participants based on the inclusion and exclusion criteria will be randomly allocated in a 1:1 ratio to either the VR training group and the non-VR training group. The attending physician will obtain the informed consent and refer the participants to the study coordinator after their baseline information has been assessed. The participants will be randomly assigned to the VR training group and non-VR training group by the study coordinator. A random allocation sequence will be generated in advance using a computerized random number generator by a biostatistician who will be not involved in this trial. The study coordinator who will be not involved in recruiting participants will keep the random list and will assign the treatment plans accordingly. The biostatistician and research coordinator will keep the random list until the end of the research to ensure that the assignment is blind to the participants and the attending physician who will recruit participants.

Although it is impossible to blind the participants and rehabilitation trainer due to this being a non-pharmacological intervention trial, the allocation will be blind to the outcome assessors and the data analyst. Participants will be instructed not to disclose the allocation to the attending physician and the outcome assessors.

### Intervention design of the VR training group and non-VR training group

The interventions of the two groups are based on the research intervention description of TIDieR template [24], shown in Table 1. Relevant concomitant care and interventions are permitted during the trial.

**Table 1:** Research interventions of the VR training group and the non-VR training group base on TIDieR template

Item	VR training group	Non-VR training group
1. Brief name	VRT	Non-VRT
2. Why	1. From the mechanism of action, VR training can induce cortical recombination of neural motor pathways; 2. From a neurological point of view, repeated training can enhance the synaptic function of the affected limb and increase the neuroplasticity induced by movement [25]; 3. From the perspective of kinematics, repetitive movement is the basic condition for acquiring a motor skill. However, to further strengthen the mastery of this skill, successful feedback and pleasant experience are needed.	
3. What materials	(1) Headsteal display (HMD) (HIC Vive Pro); (2) Vive wireless suite; (3) Vive locator, which senses the accurate position and motion of subjects by using SteamVR tracking technology, g-sensor correction, gyroscope, proximity distance sensor, and pupil distance sensor; (4) two operating handles; (5) computer configuration; (6) VR lower limb gait training scenarios for patients to choose from (nine-grid training, single-plank bridge training, climbing stairs training, and crossbar training)	Routine training equipment, lower limb training treadmill, rehabilitation training bicycle
4. Procedures	The VR training group will receive (1) 30 min per day for 3 weeks (5 days/week) of VR-assisted gait rehabilitation training. Specific process of VR training: patients wear HTC Vive virtual glasses on the open ground and are synchronously equipped with different scenes such as nine-grid training, single-plank bridge training, climbing stairs training, and crossbar training. VR scenes are immersive. Patients are placed in virtual scenes and receive corresponding scores after completing tasks according to the training requirements of different scenes. (2) 30 min per day for 3 weeks (5 days/week) of regular active exercise training (unarmed drafting, muscle strength training, trunk balance, transfer).	The non-VR training group receives (1) 30 min per day for 3 weeks (5 days/week) of functional gait rehabilitation training (standing balance training, separation promoting exercise, center of gravity training, body position change training, step separation training, parallel bar walking training). (2) 30 min per day for 3 weeks (5 days/week) of regular active exercise training (unarmed drafting, muscle strength training, trunk balance, transfer).
5. Who provided	The intervention measures of the two groups in this study were carried out by rehabilitation therapists with over 3 years of experience. Intervention measures were developed by experimental researchers.	
6. How	Both interventions in this study will be conducted in one-on-one training sessions daily.	
7. Where	The intervention will be carried out in the inpatient ward of the Department of Neurology of the union hospital affiliated with Fujian Medical University.	
8. When and how much	During 3 weeks of rehabilitation training, both groups will receive training lasting 30 min/day and 5 days/week for 3 weeks. The intensity was adjusted immediately according to the gait function of patients’ lower limbs.	
9. Tailoring	The intervention of the two groups can be adjusted according to the gait function of patients. Before training, therapists will make a simple and rapid assessment of the lower limb function of patients to select the personalized intervention intensity suitable for patients. It can also be adjusted according to patients’ own preferences.	
11. How well	Evaluators overseeing intervention results will be blinded to the group allocation process and randomization results to ensure the objectivity and impartiality of the evaluation results.	

### VR system

**Fig. 3:**
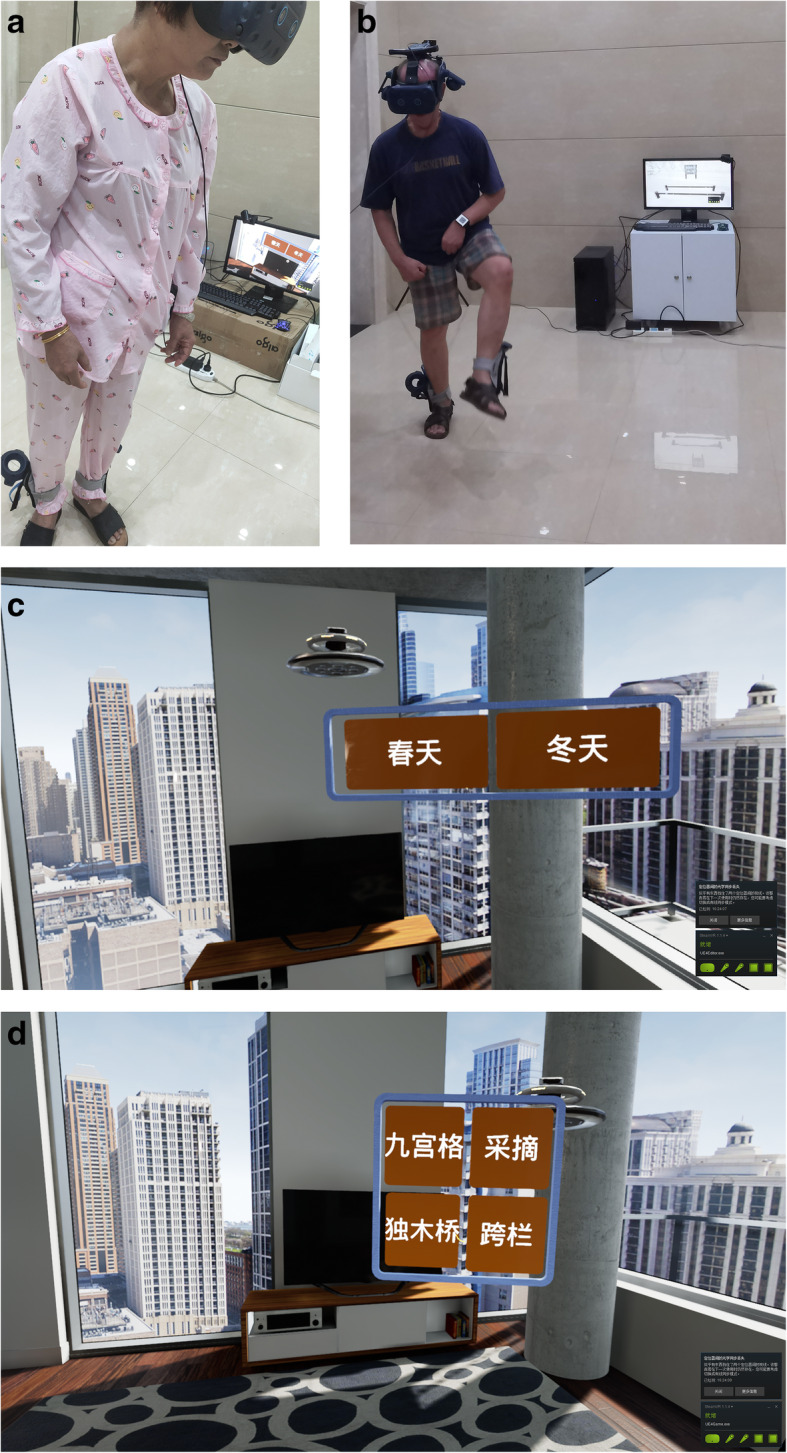
Virtual reality (VR) system and virtual scene. **a** A participant wears a virtual reality system. **b** A participant is carrying out crossbar training

**Fig. 4:**
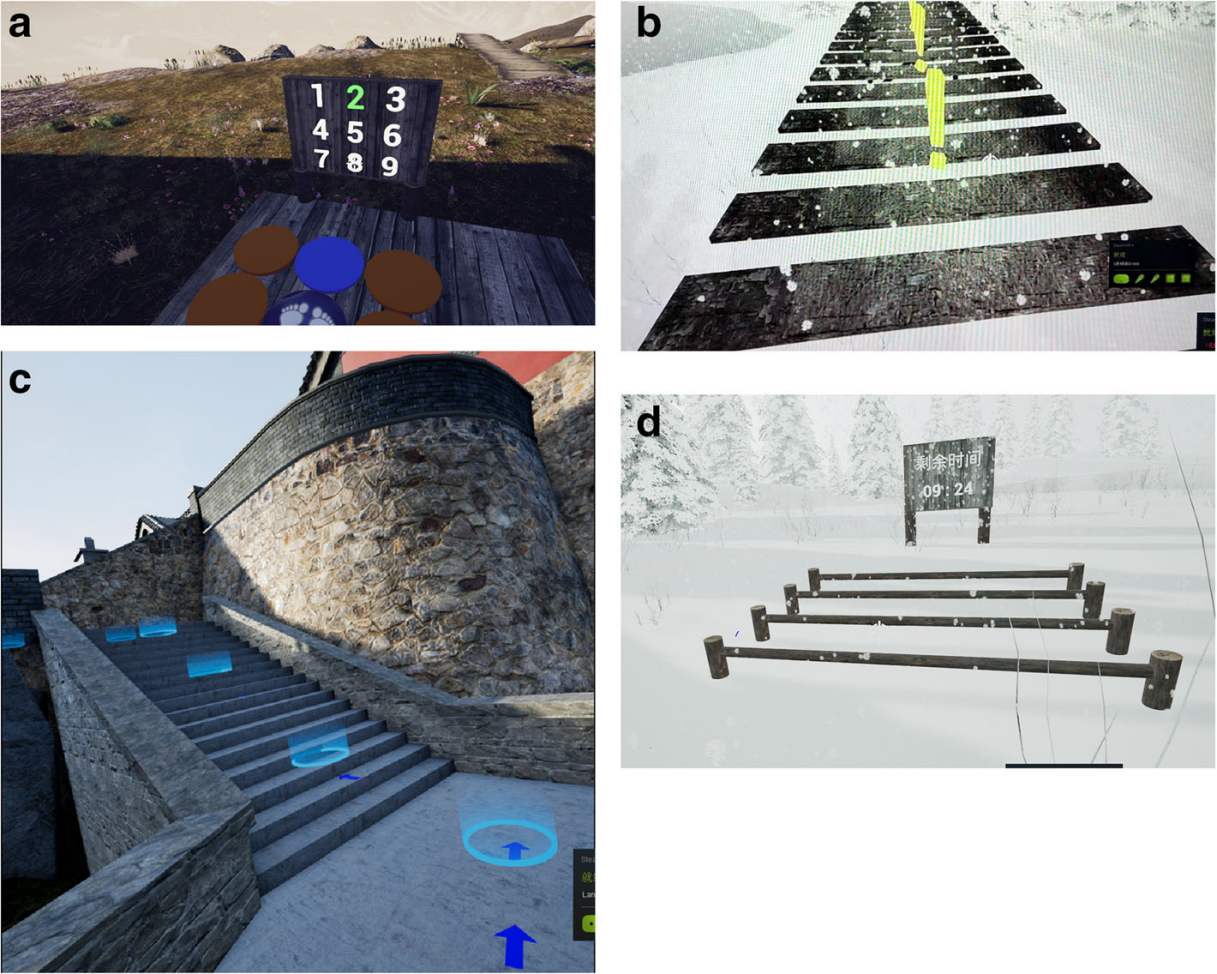
Four virtual reality training scenes. **a** Nine-grid training: participants can score points by trampling on the blue grid, and the color change speed of the grid can be adjusted. **b** Single-plank bridge training: participants can score points when they walk to the end of a single-plank bridge; the plank spacing of the bridge is adjustable. **c** Climbing stairs training: participants can score points for successful mountaineering to voice prompts. **d** Crossbar training: participants can score points when they cross the bar; the height of the bar is adjustable

As shown in Fig. 3, the VR system consists of (1) a helmet display (HMD) (HTC Vive Pro), (2) Vive wireless suite, and (3) Vive locator equipped with Steam tracking technology, g-sensor correction, gyroscope, proximity distance sensor, and pupil distance sensor to sense the accurate position and motion of the subject. The system also includes (4) two operating handles and (5) computer configuration (a computer equipped with Intel®Core™ i7-7700k (4.20GHz) quad-core processor, 32 GB random access memory, and 8 GB Nvidia GeForce GTX 1080 graphics card). (6) Four VR-assisted lower limb gait training scenarios will be available for patients to choose from (nine-grid training, single-plank bridge training, mountain climbing and climbing stairs training, and crossbar training). VR training scenarios are shown in Fig. 4.

### VR training group

The VR training group will receive 30 min per day for 3 weeks (5 days/week) of VR-assisted gait rehabilitation training. The specific process of VR training included the following: subjects will train on flat ground in an area of about 2 m × 2 m. Subjects will wear HTC Vive virtual glasses [26] with their feet bound with a handle to replace the feet of subjects in the virtual scenario. Four VR training scenes (nine-grid training, single-plank bridge training, climbing stairs training, and crossbar training) will be available for subjects to choose from. Participants will be instructed to select various options in the virtual scene by the eye gaze method (when subjects stare at an option in the scene for a certain period of time, that option is selected). The VR scene is immersive, giving the subject a realistic feeling. The subject will be able to turn their head to observe the surrounding environment, including looking down to see their moving feet on the ground. The participants will be placed in the virtual scene, and within 30 min, they will receive corresponding training scores according to task completion requirements set forth for different training scenes. The virtual training scenes can be switched according to the needs and preferences of the participants. The training difficulty of the VR scene can also be adjusted according to the participants’ lower extremity motor ability, allowing personalization of the VR experience. They also will receive 30 min per week for 3 weeks of regular active exercise training. Regular active exercise training will include unarmed drafting, muscle strength training, trunk balance, and transfer.

### Non-VR training group

Participants were randomly assigned to a n*on-VR training group*. They will receive 30 min per day for 3 weeks (5 days/week) of functional gait rehabilitation training. The functional gait rehabilitation training may include standing balance training, separation promoting exercise, center of gravity training, body position change training, step separation training, and parallel bar walking training. They also will receive 30 min per week for 3 weeks of regular active exercise training.

### Outcome measures

All participants in the study will be measured before the start of the study, after the intervention (3 weeks later), and again at a 6-month follow-up. A summary of all measurements in this trial is shown in Table 2.

**Table 2:** Outcome domains and measurement instruments

Outcome domain	Measurement instrument	Abbreviation	T0	T1	T2
**Primary outcomes**					
Functional walking ability	Time “up & go” test	TUGT	×	×	×
**Secondary outcomes**					
MMT strength	MMT muscle strength grading standard	MMT	×	×	×
Motor function of lower limbs	Fugl-Meyer scale	FMA	×	×	×
Motor function	Motor function assessment scale	MAS	×	×	×
Ability of daily life activities	Improved Barthel index scale	ADL	×	×	×
Kinematic parameters of gait	Step with maximum knee angle		×	×	×
Time parameters	Total support time, step frequency		×	×	×
Distance parameters	Step length, pace, stride length		×	×	×

*T0* baseline, *T1* post-intervention (3 weeks), *T2* follow-up (6 months post intervention)

#### Primary outcomes

##### Time “up & go” test (TUGT)

The time “up & go” test is a rapid quantitative assessment of functional walking ability. Studies have confirmed that the TUGT is a reliable, effective, and easy to operate clinical tool for assessing advanced functional range of the activity after stroke [27]. It has also been reported that the TUGT has a better correlation with stroke patients’ balance function, motor function, and ability of daily life activity. The test also boasts high sensitivity in predicting the risk of falls and daily self-care ability of patients. In addition to recording the time taken, gait and risk of falling will be rated based on the following criteria:1 point: normal, 2 points: very slight abnormality, 3 points: mild abnormality, 4 points: moderate abnormality, and 5 points: severe abnormality.

#### Secondary outcomes

##### MMT muscle strength (lower limbs)

The muscle strength grading standard is divided into 0–5 levels [28]: each level can be further subdivided by “+/−”.

Level 0: unknown muscle contraction;

Level 1: slight contraction, but no joint movement;

Level 2: in the state of weight reduction, the whole range of joints can be operated;

Level 3: can resist the full range of joint motion under heavy force, but cannot resist the resistance;

Level 4: can resist gravity and certain resistance movement; and

Level 5: anti-gravity and anti-full resistance exercise.

Used unarmed muscle strength test to grade limb muscle strength of stroke patients.

##### Lower extremity of the Fugl-Meyer scale

Fugl-Meyer is a carefully designed, feasible, and effective evaluation method which has been widely used in the assessment of recovery of stroke patients. The Fugl-Meyer exercise scale is highly recommended as a clinical and research tool for assessing changes in exercise injury after stroke [29]. As a rating scale for motor function recovery in stroke patients, its reliability, validity, and reactivity have been confirmed and widely accepted [30]. The Fugl-Meyer rating scale evaluates the motor function of the lower limbs through reflex activity, joint motor function, gait coordination ability and speed, etc.

##### Motor assessment scale (MAS) of the lower extremity of stroke patients

Many studies have demonstrated that MAS is a reliable and effective tool for measuring motor function of stroke patients by exploring its reliability, validity, and factor structure [31, 32].

##### Improved Barthel index scale

Strong evidence has supported the Barthel index as a reliable measure of basic life activity ability after stroke [33, 34]. This table is used to evaluate the ability of daily living activities (ADL), which is one of the characteristics and commonly used scales in rehabilitation medicine. The evaluation is based on the actual daily performance of patients, rather than the possible abilities of patients. Scores range from 0 to 20: very severe dysfunction, 25 to 45 points = severe dysfunction, 50 to 70 = moderate functional impairment, 75 to 95 points = mild functional impairment, and 100 points = ADL self-care.

##### Parameters of gait kinematics

The maximum knee joint angle of the step phase, time parameters (including total support time and step frequency), and distance parameters (including step length, step speed, stride length and variation coefficient, etc.). The kinematics parameters of gait will be observed through three-dimensional gait analysis. A gait analyzer (MA10 System, GYENNO, Shenzhen, China) will be used to analyze the gait kinematics parameters of the two groups.

### Sample size calculation

The sample size calculations are based on the primary outcome measure, the TUGT scale [35]. It was estimated that a sample of 28 participants (14 per group) would provide 80% power (*α* = 0.05, *β* = 0.2) to detect a difference between group means of 3.16. A difference of 3.16 points on the TUGT scale is regarded as minimum detectible change [36] using the program PASS, which takes into account the number of sample groups and the number of evaluation measures. To reach this total number of individuals, at least 36 individuals must be recruited, allowing a dropout rate of 20%.

### Statistical analysis

SPSS statistical software will be used to build and process all data collected. Paired *t* tests will be used to evaluate the differences between group rehabilitation metrics before and after rehabilitation training. An independent sample *t* test will be used for measurement data between the groups. *P* < 0.05 is considered as the standard for significant difference. In addition, covariance analysis will be performed to adjust for any significant demographic or clinical imbalances. A safety analysis is scheduled after randomization and active treatment of 18 patients. Exploratory subgroup analyses may be conducted. To prevent data loss, all questionnaires will be user-friendly and collected electronically, with all study personnel trained to identify and engage subjects most likely to drop out of subsequent evaluations.

### Study organization

This study will be organized and coordinated by researchers at the Department of Neurology of the union hospital affiliated with Fujian Medical University. Research staff will also oversee VR scene design and execution of clinically related experiments.

### Study oversight and participant confidentiality

The supervision of the project will be carried out by the safety supervision committee of the hospital, which is mainly responsible for the evaluation of the experimental design, the scientific and experimental process, the safety of participants, adherence to medical ethics, and the correct management and processing of data.

The privacy rights of each subject will be respected. All initial data and outcome indicators of participants will be kept in a highly secured database and anonymous work will be done to ensure the safety and rights of each subject.

### Safety

VR is an emerging technology and its related products have been gradually developed in recent years. To date, no relevant adverse events have been reported. In the process of training subjects’ gait recovery with VR technology; our research team will strictly follow the relevant regulations of the medical ethics committee to ensure the maximum safety and ethical protection of subjects. As VR technology is a relatively new technology, subjects may not be able to complete training tasks due to ineptness or intolerance (such as dizziness, nausea, sore eyes, disorientation) during training. Adverse events (e.g., falls, pain, and dizziness) that occur during the study period, whether or not related to the study intervention are registered. Considering the above two aspects, we will take the following measures to achieve the best training environment:

1. During the 3-week training, a strict training plan will be made for each subject on a weekly basis, adjusting the training schedule according to the actual conditions of the subjects;
2. Pay close attention to the feedback and fitness of subjects using VR technology for rehabilitation training;
3. Periodically evaluate the intervention effect of subjects using VR technology for rehabilitation training; and
4. Supporting subjects’ decision to withdraw from the study.

This study will strictly comply with the requirements of clinical operation guidelines and internationally recognized legal requirements. Any adverse events will be reported directly to the ethics committee immediately, and the training regimen will be adjusted accordingly after each adverse event. There is no anticipated harm and compensation for trial participation.

### Quality control and quality assurance

Before the start of the study, in order to ensure the quality of the research, we will strictly review the recruited doctors, rehabilitation therapists, evaluators, and VR equipment, and take the following specific measures:

1. The recruited doctors should fully understand the experimental process;
2. Train therapists to carry out VR-assisted rehabilitation training of subjects and how to deal with potential adverse events during the training;
3. Train evaluators how to appropriately manage and process outcome indicators;
4. Among the 38 subjects included, the data of the subjects who finally completed all the training programs shall be analyzed for completeness and quality; and
5. Conduct debugging of VR equipment and gait analyzer (data analysis equipment) so as to achieve the optimal running state of the equipment.

### Others

The steering committee consists of the authors Huihui Cai, Tao Lin, Lina Chen, Huidan Weng, Ruihan Zhu, Ying Chen, and Guoen Cai. All members are independent of the sponsor. The committee members participate in designing the study and supporting the on-going trial with advice. There is a data safety monitoring board which is independent of the sponsor, including statisticians and a chairman who is Prof. Qinyong Ye. An audit may be performed by the medical supervision and management department to evaluate clinical study conduct and compliance with the protocol, SOPs, GCP, and the applicable regulatory requirements.

### Protocol amendments

We will discuss the protocol amendments in the Scientific Board and notify the sponsor and funder first. A copy of the revised protocol will be sent to the PI. Any deviations from the protocol will be fully documented using a breach report form.

### Dissemination plans

The datasets analyzed during the current study are available from the corresponding author on reasonable request. Trial data and results will be published in scientific journals. The consent form and trial information materials are available from the corresponding author on request.

## Discussion

In this study, the effects of immersive VR technology on gait rehabilitation of stroke patients will be evaluated after 3 weeks of intervention and at a 6-month follow-up. The improvement of traditional gait-disorder rehabilitation for stroke patients is required as the traditional method is time- and resource-consuming and relatively one-dimensional with regard to the patient experience. The effectiveness and feasibility of the newly developed VR technology in rehabilitation training could provide key solutions to these limitations. The main purpose of the proposed study protocol is to establish an immersive VR gait rehabilitation training experience and comparatively evaluate outcomes in a controlled, randomized experiment.

VR technology leverages interactive simulations to create an environment that is similar to the real-world scenarios. Stroke patients can complete tasks as required and interact with virtual objects. Previous studies have pointed out that such human-computer interactions should be easy to adapt, be intuitive and convenient to use, and allow the subjects to readily transfer the skills learned in the virtual environment to real life. In addition, VR can provide immediate motor feedback during training, allowing for task-oriented repetitive exercises while enabling changes to the traditional treatment patterns which can feel repetitive or boring for patients. In the form of a virtual game, the treatment of patients with lively and interesting scenes and training projects allows patients to feel more relaxed, engaged, and therefore more invested in the active participation. The real-time feedback can additionally provide patients with a greater awareness of their training and performance [37]. In addition to repetitive training, the modulation of different environments during a VR-assisted training session can also be very helpful for patients’ learning ability, which can enhance the ability of patients to adapt to the environment and thus improve their sports learning ability [3, 38]. This mode of higher participation can ultimately improve the enthusiasm and acceptance of training for patients, reducing the dropout rate and enabling patients to maintain long-term rehabilitation [39]. Moreover, studies have shown that immersive VR is feasible in the rehabilitation of stroke patients with gait disorder [20]. Interestingly, one study has found that subjects receiving occasional intermittent treatment were more effectively impacted than others receiving daily treatment [40].

## Trial status

The study is actively recruiting participants. The end of the trial phase and subsequent data analysis are expected to be completed within 24 to 36 months. The protocol version number is 4.0. Recruitment started on December 1, 2019, and will close on December 1, 2022.

## Supplementary Information

**Additional file 1.** SPIRIT checklist. The table identifies the page number for the relevant content.

## Data Availability

Study data can be provided on request. For interested researchers, please contact Dr. Cai.

## Abbreviations

VR: Virtual reality
TUGT: Time “up & go” test
MAS: Motor assessment scale
HMD: Helmet display
ADL: The ability of daily living activities
